# [1-(1-Adamantylamino)ethyl­idene]oxonium methane­sulfonate

**DOI:** 10.1107/S1600536809017632

**Published:** 2009-05-20

**Authors:** Robert Vícha, Marek Nečas, Zuzana Kozubková, Milan Potáček

**Affiliations:** a Department of Chemistry, Faculty of Technology, Tomas Bata University in Zlin, Nám. T. G. Masaryka 275, Zlín,762 72, Czech Republic; b Department of Chemistry, Faculty of Science, Masaryk University in Brno, Kamenice 5, Brno-Bohunice, 625 00, Czech Republic

## Abstract

In the title salt, C_12_H_20_NO^+^·CH_3_SO_3_^−^, the [1-(1-adamantyl­amino)ethyl­idene]oxonium cations and methane­sulfonate anions are linked into chains along the *a* axis *via* O—H⋯O and N—H⋯O hydrogen bonds. All non-H atoms of the acetamido group are essentially planar, with a maximum deviation of 0.0085 (12) Å. In comparison with related structures, the carbonyl C=O bond is slightly elongated [1.249 (2) Å], whereas the amide C—N bond is shortened [1.292 (2) Å].

## Related literature

For previously published structures of *N*-(1-adamant­yl)­acetamide, see: Pröhl *et al.* (1997 ▶); Kashino *et al.* (1998 ▶); Mizoguchi *et al.* (1997 ▶). For the preparation of *N*-(1-adaman­t­yl)­acetamide, see: Bach *et al.* (1979 ▶, 1980 ▶); Gerzon *et al.* (1963 ▶); Stetter *et al.* (1959 ▶, 1960 ▶). For the biological activity of related adamantane derivatives, see: Davies *et al.* (1964 ▶); Aldrich *et al.* (1971 ▶).
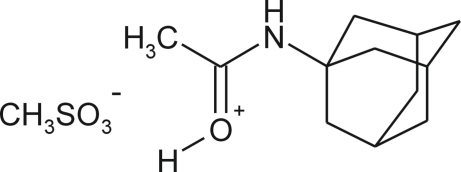


## Experimental

### 

#### Crystal data

C_12_H_20_NO^+^·CH_3_SO_3_^−^
*M* *_r_* = 289.38
Orthorhombic, 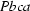

*a* = 12.9848 (7) Å
*b* = 11.2625 (6) Å
*c* = 19.0037 (10) Å
*V* = 2779.1 (3) Å^3^
*Z* = 8
Mo *K*α radiation
μ = 0.24 mm^−1^
*T* = 120 K
0.40 × 0.40 × 0.35 mm

#### Data collection

Kuma KM-4 CCD diffractometer
Absorption correction: multi-scan (*Xcalibur*; Oxford Diffraction, 2006 ▶) *T*_min_ = 0.824, *T*_max_ = 0.914
19453 measured reflections
2454 independent reflections
2000 reflections with *I* > 2σ(*I*)
*R*_int_ = 0.017

#### Refinement

*R*[*F* ^2^ > 2σ(*F* ^2^)] = 0.034
*wR*(*F* ^2^) = 0.102
*S* = 1.09
2454 reflections
180 parameters
H atoms treated by a mixture of independent and constrained refinement
Δρ_max_ = 0.30 e Å^−3^
Δρ_min_ = −0.47 e Å^−3^

### 

Data collection: *Xcalibur* (Oxford Diffraction, 2006 ▶); cell refinement: *Xcalibur* (Oxford Diffraction, 2006 ▶); data reduction: *Xcalibur* (Oxford Diffraction, 2006 ▶); program(s) used to solve structure: *SHELXS97* (Sheldrick, 2008 ▶); program(s) used to refine structure: *SHELXL97* (Sheldrick, 2008 ▶); molecular graphics: *ORTEP-3* (Farrugia, 1997 ▶); software used to prepare material for publication: *SHELXL97*.

## Supplementary Material

Crystal structure: contains datablocks I, global. DOI: 10.1107/S1600536809017632/pk2161sup1.cif

Structure factors: contains datablocks I. DOI: 10.1107/S1600536809017632/pk2161Isup2.hkl

Additional supplementary materials:  crystallographic information; 3D view; checkCIF report

## Figures and Tables

**Table 1:** Hydrogen-bond geometry (Å, °)

*D*—H⋯*A*	*D*—H	H⋯*A*	*D*⋯*A*	*D*—H⋯*A*
N1—H1⋯O3^i^	0.87 (2)	1.98 (2)	2.838 (2)	170.1 (19)
O1—H2⋯O2	0.98 (2)	1.49 (2)	2.4632 (18)	173 (2)

Symmetry code: (i) 
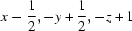
.

**Fig. 1.**
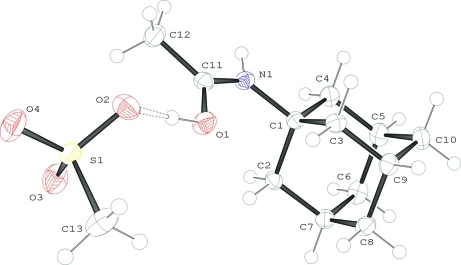
ORTEP of the asymmetric unit with atoms represented as 50% probability ellipsoids. Hydrogen bonding is indicated by dashed lines.

**Fig. 2.**
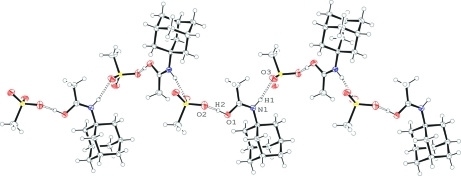
Hydrogen bonded chains of alternating protonated N-(1-adamantyl)acetamide and methansulfonate parallel to the a axis.

## supplementary crystallographic information

### Comment

Since 1964, 1-aminoadamantane and related compounds have seen extensive examination due to their antiviral activity (Davies *et al.*, 1964; Aldrich *et al.*, 1971). The consecution of adamantane bromination, reaction with acetonitrile and final hydrolysis of *N*-(1-adamantyl)acetamide provides a viable synthetic method for 1-aminoadamantane production. The synthesis of *N*-(1-adamantyl)acetamide *via* nucleophilic substitution from varied bridgehead-substituted derivatives was previously described. For this purpose 1-iodoadamantane (Bach *et al.*, 1980), 1-bromoadamantane (Stetter *et al.*, 1960), 1-chloroadamantane (Gerzon *et al.*, 1963), 1-alkoxyadamantane (Bach *et al.*, 1979; Bach *et al.*, 1980) or adamantan-1-ol (Stetter *et al.*, 1959) were used as starting material. The title salt was prepared by replacement of a good-leaving group in 1-adamantyl methanesulfonate with acetonitrile.

In the structure of title salt (Fig. 1), the *O*-protonated *N-*(1-adamantyl)acetamide and methansulfonate are linked alternately into chains parallel to the *a* axis *via* O1–H2···O2 and N1–H1···O3 hydrogen bonds (Table 1, Fig. 2). All non-hydrogen atoms of the acetamido group (C11, C12, N1, O1) and C1 lie in plane with the maximum deviation from the best plane being 0.0085 (12) Å for atom N1. The distance of the H1 and H2 from the best plane (C1, C11, C12, N1, O1) is 0.004 (19) and 0.11 (2) Å respectively. In comparison with previously published structures of *N*-(1-adamantyl)acetamide (Pröhl *et al.*, 1997; Kashino *et al.*, 1998 and Mizoguchi *et al.* , 1997), the length of N1–C11 is slightly shorter, being 1.292 (2) Å [published 1.323 (5)–1.345 (2) Å] and C11–O1 is slightly longer at 1.294 (2) Å [published 1.230 (5)–1.237 (4) Å]. This may be attributed to enhanced electron withdrawing effect of the protonated O1.

### Experimental

1-Adamantyl methansulfonate (500 mg, 2.17 mmol) was stirred in 20 ml of dry acetonitrile at room temperature for 1 h. After this period, the solution was allowed to stand at room temperature for several days and growth of crystals was observed. The solid was filtered off with suction and mother liquor was evaporated to obtain a second crop of title compound as a colourless powder. The combined yield of the title salt was 583 mg (93%). ^1^H NMR spectra were similar to those obtained for equimolar mixture of separately prepared *N*-(1-adamantyl)acetamide and methanesulfonic acid.

### Refinement

H atoms were found in difference Fourier maps.  Those attached to N and O were refined while those attached to C were placed in idealized positions with constrained distances of 0.98 Å (RCH_3_) and 0.99 Å (R_2_CH_2_). U_iso_(H) values were set to either 1.2U_eq_ or 1.5U_eq_ (RCH_3_, OH) of the attached atom.

### Crystal data

**Table:**

C_12_H_20_NO^+^·CH_3_SO_3_^−^	*D*_x_ = 1.383 Mg m^−^^3^
*M**_r_* = 289.38	Melting point: 445 K
Orthorhombic, *P**b**c**a*	Mo *K*α radiation, λ = 0.71073 Å
Hall symbol: -P 2ac 2ab	Cell parameters from 2454 reflections
*a* = 12.9848 (7) Å	θ = 3.1–25.0°
*b* = 11.2625 (6) Å	µ = 0.24 mm^−^^1^
*c* = 19.0037 (10) Å	*T* = 120 K
*V* = 2779.1 (3) Å^3^	Block, colourless
*Z* = 8	0.40 × 0.40 × 0.35 mm
*F*(000) = 1248	

### Data collection

**Table:**

Kuma KM-4 CCD diffractometer	2454 independent reflections
Radiation source: fine-focus sealed tube	2000 reflections with *I* > 2σ(*I*)
graphite	*R*_int_ = 0.017
Detector resolution: 0.06 pixels mm^-1^	θ_max_ = 25.0°, θ_min_ = 3.1°
ω scans	*h* = −15→14
Absorption correction: multi-scan (Xcalibur; Oxford Diffraction, 2006)	*k* = −13→13
*T*_min_ = 0.824, *T*_max_ = 0.914	*l* = −21→22
19453 measured reflections	

### Refinement

**Table:**

Refinement on *F*^2^	Primary atom site location: structure-invariant direct methods
Least-squares matrix: full	Secondary atom site location: difference Fourier map
*R*[*F*^2^ > 2σ(*F*^2^)] = 0.034	Hydrogen site location: inferred from neighbouring sites
*wR*(*F*^2^) = 0.102	H atoms treated by a mixture of independent and constrained refinement
*S* = 1.09	*w* = 1/[σ^2^(*F*_o_^2^) + (0.054*P*)^2^ + 1.529*P*] where *P* = (*F*_o_^2^ + 2*F*_c_^2^)/3
2454 reflections	(Δ/σ)_max_ = 0.001
180 parameters	Δρ_max_ = 0.30 e Å^−^^3^
0 restraints	Δρ_min_ = −0.47 e Å^−^^3^

### Special details

**Table:**

Geometry. All e.s.d.'s (except the e.s.d. in the dihedral angle between two l.s. planes) are estimated using the full covariance matrix. The cell e.s.d.'s are taken into account individually in the estimation of e.s.d.'s in distances, angles and torsion angles; correlations between e.s.d.'s in cell parameters are only used when they are defined by crystal symmetry. An approximate (isotropic) treatment of cell e.s.d.'s is used for estimating e.s.d.'s involving l.s. planes.
Refinement. Refinement of *F*^2^ against ALL reflections. The weighted *R*-factor *wR* and goodness of fit *S* are based on *F*^2^, conventional *R*-factors *R* are based on *F*, with *F* set to zero for negative *F*^2^. The threshold expression of *F*^2^ > 2σ(*F*^2^) is used only for calculating *R*-factors(gt) *etc*. and is not relevant to the choice of reflections for refinement. *R*-factors based on *F*^2^ are statistically about twice as large as those based on *F*, and *R*- factors based on ALL data will be even larger.

### Fractional atomic coordinates and isotropic or equivalent isotropic displacement parameters (Å^2^)

**Table:**

	*x*	*y*	*z*	*U*_iso_*/*U*_eq_	
C1	−0.09044 (13)	0.28700 (14)	0.34406 (9)	0.0181 (4)	
C2	−0.00380 (13)	0.22857 (15)	0.30176 (9)	0.0195 (4)	
H2A	0.0621	0.2698	0.3111	0.023*	
H2B	0.0038	0.1445	0.3162	0.023*	
C3	−0.10319 (14)	0.41701 (15)	0.32215 (9)	0.0201 (4)	
H3A	−0.1594	0.4541	0.3496	0.024*	
H3B	−0.0388	0.4613	0.3317	0.024*	
C4	−0.19133 (14)	0.22087 (16)	0.33012 (9)	0.0229 (4)	
H4A	−0.1846	0.1370	0.3451	0.027*	
H4B	−0.2477	0.2576	0.3576	0.027*	
C5	−0.21663 (15)	0.22635 (17)	0.25177 (10)	0.0251 (4)	
H5	−0.2824	0.1828	0.2427	0.030*	
C6	−0.13002 (15)	0.16913 (16)	0.20928 (10)	0.0262 (4)	
H6A	−0.1467	0.1724	0.1585	0.031*	
H6B	−0.1225	0.0847	0.2230	0.031*	
C7	−0.02922 (14)	0.23530 (16)	0.22322 (10)	0.0225 (4)	
H7	0.0276	0.1979	0.1954	0.027*	
C8	−0.04113 (14)	0.36523 (15)	0.20149 (9)	0.0224 (4)	
H8A	−0.0567	0.3702	0.1506	0.027*	
H8B	0.0240	0.4084	0.2103	0.027*	
C9	−0.12838 (13)	0.42199 (15)	0.24371 (9)	0.0213 (4)	
H9	−0.1362	0.5068	0.2291	0.026*	
C10	−0.22900 (14)	0.35595 (17)	0.22953 (10)	0.0256 (4)	
H10A	−0.2461	0.3603	0.1788	0.031*	
H10B	−0.2858	0.3932	0.2564	0.031*	
C11	0.00322 (13)	0.32640 (15)	0.45588 (9)	0.0211 (4)	
C12	0.01287 (15)	0.30548 (18)	0.53284 (10)	0.0271 (4)	
H12A	0.0762	0.2609	0.5424	0.041*	
H12B	−0.0467	0.2599	0.5495	0.041*	
H12C	0.0155	0.3819	0.5575	0.041*	
N1	−0.06903 (11)	0.27539 (13)	0.42026 (8)	0.0195 (3)	
H1	−0.1102 (15)	0.2308 (18)	0.4446 (11)	0.023*	
O1	0.06855 (10)	0.39456 (12)	0.42407 (6)	0.0268 (3)	
H2	0.1158 (17)	0.436 (2)	0.4560 (12)	0.040*	
S1	0.29158 (4)	0.49446 (4)	0.50332 (2)	0.02087 (16)	
O2	0.17962 (12)	0.51334 (12)	0.50208 (7)	0.0302 (3)	
O3	0.31512 (12)	0.37147 (12)	0.48779 (7)	0.0342 (4)	
O4	0.33796 (11)	0.54034 (13)	0.56618 (7)	0.0357 (4)	
C13	0.33885 (17)	0.57840 (19)	0.43323 (10)	0.0356 (5)	
H13A	0.3191	0.6617	0.4396	0.053*	
H13B	0.3099	0.5485	0.3890	0.053*	
H13C	0.4141	0.5721	0.4318	0.053*	

### Atomic displacement parameters (Å^2^)

**Table:**

	*U*^11^	*U*^22^	*U*^33^	*U*^12^	*U*^13^	*U*^23^
C1	0.0179 (9)	0.0183 (8)	0.0179 (9)	0.0002 (7)	0.0006 (7)	0.0014 (7)
C2	0.0182 (9)	0.0204 (9)	0.0201 (9)	0.0034 (7)	−0.0005 (7)	−0.0017 (7)
C3	0.0235 (9)	0.0163 (8)	0.0206 (9)	0.0009 (7)	0.0028 (7)	−0.0011 (7)
C4	0.0213 (9)	0.0212 (9)	0.0261 (10)	−0.0034 (7)	−0.0001 (7)	0.0048 (7)
C5	0.0213 (9)	0.0250 (10)	0.0289 (10)	−0.0074 (8)	−0.0056 (7)	0.0029 (8)
C6	0.0341 (11)	0.0185 (9)	0.0259 (10)	−0.0019 (8)	−0.0061 (8)	−0.0019 (7)
C7	0.0250 (9)	0.0230 (9)	0.0195 (9)	0.0043 (8)	−0.0002 (7)	−0.0037 (7)
C8	0.0238 (9)	0.0260 (9)	0.0176 (9)	−0.0027 (8)	−0.0004 (7)	0.0002 (7)
C9	0.0254 (9)	0.0150 (8)	0.0236 (9)	0.0005 (7)	−0.0008 (7)	0.0027 (7)
C10	0.0209 (9)	0.0281 (10)	0.0277 (10)	0.0022 (8)	−0.0044 (8)	0.0042 (8)
C11	0.0207 (9)	0.0209 (9)	0.0216 (9)	0.0041 (7)	0.0029 (7)	−0.0015 (7)
C12	0.0311 (11)	0.0299 (10)	0.0202 (10)	0.0010 (8)	−0.0021 (8)	0.0001 (8)
N1	0.0214 (8)	0.0187 (7)	0.0183 (8)	−0.0001 (6)	0.0022 (6)	0.0015 (6)
O1	0.0257 (7)	0.0330 (7)	0.0216 (7)	−0.0080 (6)	−0.0009 (5)	−0.0008 (6)
S1	0.0221 (3)	0.0229 (3)	0.0175 (3)	−0.00163 (17)	−0.00125 (16)	0.00108 (17)
O2	0.0240 (7)	0.0328 (8)	0.0337 (8)	−0.0003 (6)	0.0006 (5)	−0.0114 (6)
O3	0.0433 (9)	0.0270 (8)	0.0323 (8)	0.0092 (6)	−0.0058 (6)	0.0028 (6)
O4	0.0406 (8)	0.0440 (8)	0.0226 (7)	−0.0109 (7)	−0.0093 (6)	0.0014 (6)
C13	0.0489 (13)	0.0353 (11)	0.0227 (10)	−0.0117 (10)	0.0030 (9)	0.0021 (8)

### Geometric parameters (Å, °)

**Table:**

C1—N1	1.480 (2)	C8—H8A	0.9900
C1—C4	1.530 (2)	C8—H8B	0.9900
C1—C3	1.531 (2)	C9—C10	1.527 (2)
C1—C2	1.531 (2)	C9—H9	1.0000
C2—C7	1.530 (3)	C10—H10A	0.9900
C2—H2A	0.9900	C10—H10B	0.9900
C2—H2B	0.9900	C11—N1	1.292 (2)
C3—C9	1.527 (2)	C11—O1	1.294 (2)
C3—H3A	0.9900	C11—C12	1.487 (3)
C3—H3B	0.9900	C12—H12A	0.9800
C4—C5	1.526 (3)	C12—H12B	0.9800
C4—H4A	0.9900	C12—H12C	0.9800
C4—H4B	0.9900	N1—H1	0.87 (2)
C5—C6	1.527 (3)	O1—H2	0.98 (2)
C5—C10	1.528 (2)	S1—O4	1.4343 (14)
C5—H5	1.0000	S1—O3	1.4489 (14)
C6—C7	1.529 (3)	S1—O2	1.4695 (15)
C6—H6A	0.9900	S1—C13	1.7448 (19)
C6—H6B	0.9900	C13—H13A	0.9800
C7—C8	1.528 (2)	C13—H13B	0.9800
C7—H7	1.0000	C13—H13C	0.9800
C8—C9	1.528 (2)		
			
N1—C1—C4	106.69 (13)	C7—C8—H8A	109.8
N1—C1—C3	111.76 (14)	C9—C8—H8A	109.8
C4—C1—C3	109.01 (14)	C7—C8—H8B	109.8
N1—C1—C2	109.73 (14)	C9—C8—H8B	109.8
C4—C1—C2	109.20 (14)	H8A—C8—H8B	108.2
C3—C1—C2	110.34 (14)	C3—C9—C10	109.73 (14)
C7—C2—C1	109.40 (14)	C3—C9—C8	109.77 (14)
C7—C2—H2A	109.8	C10—C9—C8	109.74 (14)
C1—C2—H2A	109.8	C3—C9—H9	109.2
C7—C2—H2B	109.8	C10—C9—H9	109.2
C1—C2—H2B	109.8	C8—C9—H9	109.2
H2A—C2—H2B	108.2	C9—C10—C5	109.05 (14)
C9—C3—C1	108.89 (14)	C9—C10—H10A	109.9
C9—C3—H3A	109.9	C5—C10—H10A	109.9
C1—C3—H3A	109.9	C9—C10—H10B	109.9
C9—C3—H3B	109.9	C5—C10—H10B	109.9
C1—C3—H3B	109.9	H10A—C10—H10B	108.3
H3A—C3—H3B	108.3	N1—C11—O1	119.67 (16)
C5—C4—C1	109.48 (14)	N1—C11—C12	120.42 (16)
C5—C4—H4A	109.8	O1—C11—C12	119.90 (16)
C1—C4—H4A	109.8	C11—C12—H12A	109.5
C5—C4—H4B	109.8	C11—C12—H12B	109.5
C1—C4—H4B	109.8	H12A—C12—H12B	109.5
H4A—C4—H4B	108.2	C11—C12—H12C	109.5
C4—C5—C6	109.89 (15)	H12A—C12—H12C	109.5
C4—C5—C10	109.34 (15)	H12B—C12—H12C	109.5
C6—C5—C10	109.53 (15)	C11—N1—C1	127.58 (15)
C4—C5—H5	109.4	C11—N1—H1	115.2 (14)
C6—C5—H5	109.4	C1—N1—H1	117.2 (14)
C10—C5—H5	109.4	C11—O1—H2	113.8 (13)
C5—C6—C7	109.45 (14)	O4—S1—O3	115.18 (9)
C5—C6—H6A	109.8	O4—S1—O2	112.12 (8)
C7—C6—H6A	109.8	O3—S1—O2	110.11 (8)
C5—C6—H6B	109.8	O4—S1—C13	107.03 (9)
C7—C6—H6B	109.8	O3—S1—C13	106.76 (9)
H6A—C6—H6B	108.2	O2—S1—C13	104.92 (10)
C8—C7—C6	109.47 (15)	S1—C13—H13A	109.5
C8—C7—C2	109.44 (14)	S1—C13—H13B	109.5
C6—C7—C2	109.23 (15)	H13A—C13—H13B	109.5
C8—C7—H7	109.6	S1—C13—H13C	109.5
C6—C7—H7	109.6	H13A—C13—H13C	109.5
C2—C7—H7	109.6	H13B—C13—H13C	109.5
C7—C8—C9	109.49 (14)		

### Hydrogen-bond geometry (Å, °)

**Table:**

*D*—H···*A*	*D*—H	H···*A*	*D*···*A*	*D*—H···*A*
N1—H1···O3^i^	0.87 (2)	1.98 (2)	2.838 (2)	170.1 (19)
O1—H2···O2	0.98 (2)	1.49 (2)	2.4632 (18)	173 (2)

Symmetry codes: (i) *x*−1/2, −*y*+1/2, −*z*+1.
